# Adverse outcomes of chronic widespread pain and common mental disorders in individuals with sickness absence – a prospective study of Swedish twins

**DOI:** 10.1186/s12889-020-09407-9

**Published:** 2020-08-27

**Authors:** Mo Wang, Annina Ropponen, Jurgita Narusyte, Björg Helgadóttir, Gunnar Bergström, Victoria Blom, Pia Svedberg

**Affiliations:** 1 Division of Insurance Medicine, Department of Clinical Neuroscience, Karolinska Institutet, SE-171 77 Stockholm, Sweden; 2 Finnish Institute of Occupational Health, Helsinki, Finland; 3 The Swedish School of Sport and Health Sciences, Stockholm, Sweden; 4 Unit of Intervention and Implementation Research for Worker Health, Institute of Environmental Medicine, Karolinska Institutet, Stockholm, Sweden; 5 Centre for Musculoskeletal Research, Department of Occupational Health Sciences and Psychology, University of Gävle, Gävle, Sweden

**Keywords:** Chronic widespread pain, Common mental disorders, Sick leave, Disability pension, Mortality, Twin study

## Abstract

**Background:**

Chronic widespread pain (CWP) and common mental disorders (CMDs) are common public health problems, but little is known about the role of CWP and CMDs on future adverse outcomes among work disabled individuals. The aims of the study were to investigate the associations between CWP and CMDs with subsequent disability pension (DP), long-term unemployment (> 90 days) and all-cause mortality in individuals with sickness absence (SA) and whether the associations were explained by familial factors.

**Methods:**

In this prospective cohort study, 7884 Swedish twins born between 1933 and 1985 were included and baseline data were gathered from a questionnaire in 1998 to 2006. Register data were used for obtaining information regarding demographics, SA, DP, unemployment and mortality. Cox proportional hazards regressions were used to calculate Hazard Ratios (HR) with 95% Confidence Intervals (CI) for the associations between CWP and/or CMDs with DP, unemployment and mortality, while conditional Cox models for twin pairs provided control for familial confounding.

**Results:**

Having either CWP or CMDs among those with a history of SA was associated with a higher risk of DP and all-cause mortality than individuals without CWP and CMDs after controlling for socio-demographic and health factors. Moreover, sick-listed individuals with both CWP and CMDs had a higher risk of DP while those who only had CMDs had a higher risk of long-term unemployment compared to those without CWP and CMDs. The association between CMDs with DP and long-term unemployment was no longer significant when controlling for familial factors.

**Conclusions:**

CMDs was a risk factor for DP, unemployment and mortality among individuals with SA, while CWP seems to be important in relation to future DP and mortality. Familial factors played a role in the associations between CMDs and DP and CMDs and unemployment.

## Background

Chronic widespread pain (CWP) is a complex and common syndrome with an estimated prevalence between 10 and 15% in the general population [1]. Individuals with CWP often experience long-lasting pain in multiple body regions and this pain is associated with other physical symptoms such as fatigue, concentration problems, and psychological distress [2, 3]. The most common reason for individuals with a complaint of CWP is fibromyalgia, but this pain complaint can be a symptom of other diseases than fibromyalgia, indicating consideration of a differential diagnosis [2]. Previous studies have shown increased mortality among individuals with CWP [4]. Also, as a disabling condition CWP might cause temporary and permanent work disability in terms of sickness absence (SA) and disability pension (DP). CWP, SA and DP are associated with a high economic and social burden for both the patients and the health care system which illustrates the necessity of further knowledge on the associations between CWP and work disability.

Another major public health problem is common mental disorders (CMDs), which are defined as mental conditions that lead to noticeable emotional distress and restrict daily function [5]. CMDs includes depression and anxiety disorders and the lifetime prevalence of these disorders is around 14% [5]. A number of studies have reported that CWP is associated with an increased prevalence of CMDs [6–8]. For example, major depressive disorder among patients with fibromyalgia is more common than in individuals without fibromyalgia [7, 8]. Also, CWP and CMDs share common symptomatology such as sleep disturbance [9], depressed mood [8], and functional impairment [10]. Nonetheless, CWP and CMDs are less frequently diagnosed and treated as comorbid conditions [8]. Some previous findings suggest an independent effect of CWP and CMDs on functional outcomes such as labour market marginalisation in terms of temporary or permanent work incapacity (that is SA and DP) or unemployment [7]. Although musculoskeletal and mental disorders are the two most common diagnosis groups behind SA in the industrialised countries [11], currently there is considerable uncertainty of the role of CWP and CMDs on future adverse outcomes in terms of permanent work disability, that is DP, unemployment, as well as mortality among individuals with SA.

Moreover, genetic factors are potentially confounding the associations between CWP and CMDs and adverse outcomes. Studies have shown genetic influences on both CWP and CMDs, with heritability estimates from twin studies for CWP, major depressive disorders and anxiety disorders as high as 71, 37 and 32%, respectively [12–14]. In addition, it has been reported that the associations between CWP and most comorbidities were influenced by unmeasured familial (genetic and shared environmental) factors [15]. Further, from previous studies we also know that SA and DP are moderately influenced by genetic factors [16, 17]. Hence, there is a possibility that familial factors influence the associations between CWP and CMDs with future SA and DP. To our knowledge, few studies have had access to twin data to examine adverse outomes of CWP and CMDs among individuals with a history of SA [18]. By studying twins that share 50–100% of their genetic material and rearing environment a possibility is provided to adjust for those unmeasured factors. Hence, more knowledge about whether familial factors influence the associations between CWP, CMDs and adverse outcomes among individuals with SA is warranted.

The aims of this study were to examine the associations between CWP and CMDs (measured combined and separately) with subsequent DP, unemployment more than 90 days and all-cause mortality in individuals with a history of SA and to investigate if the associations were explained by familial factors, using a twin design.

## Methods

### Sample and data

This prospective twin cohort study was based on data from the Swedish Twin project of Disability pension and Sickness absence (STODS). Data from the following sources were included, which were merged using the unique personal identification number of participants:

1. The Swedish Twin Registry (STR): A population-based registry that contains almost all twins born in Sweden since 1886 [19].
2. Statistics Sweden’s Longitudinal Integration Database for Health Insurance and Labour Market Studies (LISA) that includes demographic and unemployment information for all Swedish residents from 1990 onwards [20].
3. The Social Insurance Agency’s MicroData for Analysis of the Social Insurance database (MiDAS) that contains dates for SA (> 14 days) and DP from 1994 and onwards.
4. The National Board of Health and Welfare’s Cause of Death Register that contains date and cause of death from 1961 and onwards.

Data from two surveys conducted by the STR were used: Screening Across the Lifespan Twin Study (SALT) [21], performed 1998–2003, and the Study of Twin Adults - Genes and Environment (STAGE) [22], conducted 2005–2006. SALT included interview data collected from all twins in the STR born before 1958 while STAGE was an extensive web-based questionnaire sent to all twins in the STR born between 1959 and 1985. The date that each participant answered the questionnaire/interview was set as the baseline.

**Fig. 1:**
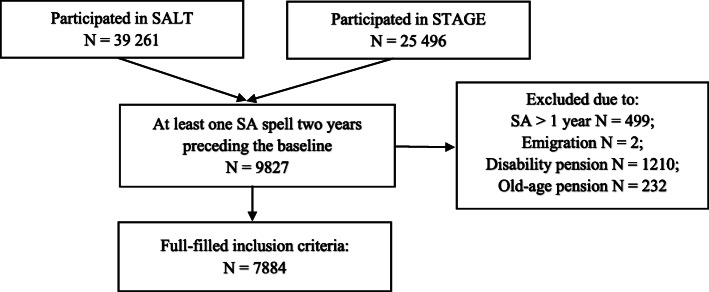
Flow chart for the study population. SA = sickness absence

Individuals who had at least one sick-leave spell due to any cause during the 2 years preceding the baseline were included in the study (*n* = 9827). Individuals who had a sick-leave spell longer than 1 year, had emigrated, were on DP or old-age pension at baseline were excluded (*n* = 1943) (Fig. 1). The final cohort contained 7884 twins (aged between 20 and 66 years, 65% women), including 1178 complete twin pairs, 466 monozygotic (MZ) pairs, 400 same-sex dizygotic (DZ), and 298 opposite sex DZ. In the sample, 162 pairs were discordant for CWP and CMDs.

### Exposure

Information on CWP and CMDs was collected in the SALT and STAGE surveys. The questions were based on a screening algorithm for CWP according to the classification criteria for fibromyalgia proposed by the American College of Rheumatology [3]. However, the difference was that our definition of CWP was based on self-reports without clinical evaluations by physicians or inspection of medical records, and that a count of tender points was not included. In the questionnaire and interview data, individuals who responded “yes” on all four of the following questions were defined as having CWP [15]: 1) “Have you suffered from general pain during the last three months?” 2) “Did you have continuous pain during all three months?” 3) “Do you suffer from pain in both the upper and lower body?” and 4) “Do you suffer from pain in both the right and left sides?”. CMDs was measured by the Diagnostic and Statistical Manual of Mental Disorders, Fourth Edition (DSM-IV) [23] major depression and anxiety. For a more detailed description of criteria for major depression and anxiety, see Mather et al. [24]. A categorical variable which combined CWP and CMDs was created and used as exposure variable: 0 = No CWP or CMDs (reference group), 1 = No CWP but having CMDs, 2 = Having CWP but no CMDs, 3 = Having both CWP and CMDs.

### Outcomes

The participants were followed up for all-cause DP, long-term unemployment (> 90 days) and all-cause mortality from baseline until the 31st of December 2012.

### Covariates

Information on sex (women/men), age (continuous variable), and zygosity (MZ, DZ) were obtained from STR. Data on education (elementary, secondary or higher education), having children under 18 living at home (yes/no), type of living area (urban or semi-urban/rural) at the end of the year of baseline was obtained from LISA. Self-rated health (excellent, good, moderate, fairly poor/poor), pain (pain in neck, shoulder or low back) (yes/no), migraine (yes/no), headache (yes/no), cancer (yes/no) at baseline was collected from the survey data. The cohort effect was binary i.e. belonging to either SALT or STAGE.

### Social insurance system in Sweden

In general, all individuals aged 16 or older with an income from work or unemployment benefits, who have a reduced work capacity due to disease or injury, can be granted sickness benefits [25]. For employees this is paid by the employer during the first 14 days, and by the Social Insurance Agency thereafter. Hence, national register data covers SA lasting more than 14 days. There is one qualifying day (more for self-employed) without benefits. All residents with a permanently impaired work capacity due to disease or injury can be granted disability pension. SA benefits covers up to 80% of lost income, DP to approximately 65%.

### Statistical analyses

Cox proportional hazards regression was performed to estimate the associations between CWP and CMDs and DP, long-term unemployment and mortality separately in the whole sample. The proportional hazards assumption was explored via graphs and was met in all the models (See supplementary materials). The follow-up started after the baseline until the end of follow-up 31st of December 2012. Follow-up (time in days) was censored for emigration and the respective outcomes. In the analysis of unemployment as outcome, DP and old-age pension were censored while in the analysis of DP, old-age pension was censored. Besides the crude model, the multivariate model was adjusted for sex, age, cohort effect, education, having children under 18 living at home, type of living area, pain in neck, shoulder or low back, migraine, headache, and cancer. As pain in neck, shoulder or low back, migraine and cancer were highly correlated with CWP, sensitivity analyses were conducted where individuals with those disorders were excluded from the analyses.

Co-twin analyses were performed separately for each outcome on exposure discordant twin pairs (i.e. one twin in a pair had CWP and/or CMDs and the other twin did not) using conditional Cox proportional hazards models. As twins are matched on genetics (100% for MZ twins and on average 50% for DZ twins) and shared environment (100% for both MZ and DZ) while growing up (if raised together), the co-twin analysis adjusts for these factors. If the association found in the age and sex adjusted analyses of the whole sample disappears or becomes weaker in the analyses of discordant twin pairs there is a suggestion that familial factors are influencing the association. If an association is still observed after controlling for familial factors this would instead suggest a direct link between the exposure and the outcome [26]. The analyses were based on discordant twin pairs (*n* = 162) for the exposure where one twin in a pair had CWP and/or CMDs and the twin sibling had neither CWP nor CMDs. MZ and DZ twin pairs were pooled for the analyses as there was not enough power to stratify by zygosity.

## Results

Frequencies of socio-demographic factors, cohort effect, health-related factors, CWP, CMDs, DP, unemployment, and mortality in the whole sample and for discordant twin pairs with a history of SA are presented in Table 1. In the whole sample, most of the participants (80.4%) had SA < 90 days. During the follow-up, 15.8% of the individuals with a history of SA had DP, 18.4% had unemployment > 90 days and 5.3% died. Among the discordant co-twins, a higher proportion of those with CWP and/or CMDs had lower educational level, reported ever having pain in neck, shoulder or low back and poorer self-rated health at baseline compared to their unaffected co-twins.

**Table 1:** Frequencies of socio-demographic factors, cohort effect, health-related factors, CWP, CMDs, SA, DP, unemployment, and mortality among 7884 twins and 162 (324 individuals) twin pairs discordant for exposure

	Whole sample	Discordant co-twins	
		Among those with CWP and/or CMDs	Among those without CWP and CMDs
	n (%)	n (%)	n (%)
**N**	7884 (100)	162 (100)	162 (100)
**Cohort**			
SALT	5072 (64.3)	102 (63.0)	102 (63.0)
STAGE	2812 (35.7)	60 (37.0)	60 (37.0)
**Sick-leave days**			
< 90 days	6338 (80.4)	128 (79.0)	123 (75.9)
90–180 days	957 (12.1)	19 (11.7)	22 (13.6)
> 180 days	589 (7.5)	15 (9.3)	17 (10.5)
**Sex**			
Men	2790 (35.4)	32 (19.8)	46 (28.4)
Women	5094 (64.6)	130 (80.3)	116 (71.6)
**Age (Mean, SD)**	46.8 (10.5)	46.2 (9.7)	46.2 (9.7)
**Zygosity**			
Monozygotic	2298 (29.2)	71 (43.8)	71 (43.8)
Dizygotic same sex	2715 (34.4)	51 (31.5)	51 (31.5)
Dizygotic opposite sex	2727 (34.6)	2 (1.2)	2 (1.2)
Unknown zygosity	144 (1.8)	38 (23.5)	38 (23.5)
**Education**			
Elementary	1647 (20.9)	32 (19.8)	24 (14.8)
Secondary	4140 (52.5)	90 (55.6)	98 (60.5)
Higher education	2093 (26.6)	40 (24.7)	40 (24.7)
Missing	4 (0.1)	0 (0)	0 (0)
**Children < 18 living at home**	3048 (38.7)	70 (43.2)	71 (43.8)
**Type of living area**			
Urban	4110 (52.1)	72 (44.4)	72 (44.4)
Semi-urban/Rural	3771 (47.8)	90 (55.6)	90 (55.6)
Missing	3 (0.04)	0 (0)	0 (0)
**Self-rated health**			
Excellent	1740 (22.1)	26 (16.1)	34 (21.0)
Good	1888 (24.0)	36 (22.2)	32 (19.8)
Moderate	654 (8.3)	20 (12.4)	18 (11.1)
Fairly poor/poor	1343 (17.0)	26 (16.1)	25 (15.4)
Missing	2259 (28.7)	54 (33.3)	53 (32.7)
**Pain**			
Back	2220 (28.2)	52 (32.1)	46 (28.4)
Neck	729 (9.3)	29 (17.9)	20 (12.4)
Shoulder	1416 (18.0)	46 (28.4)	30 (18.5)
**Headache**	1999 (25.4)	56 (34.6)	46 (28.4)
**Migraine**	856 (10.9)	27 (16.7)	24 (14.8)
**Cancer**	260 (3.3)	4 (2.5)	7 (3.2)
**CWP and/or CMDs**			
No CWP and CMDs	6447 (81.8)	0 (0)	162 (100)
Having CWP but no CMDs	374 (4.7)	40 (24.7)	0 (0)
No CWP but having CMDs	947 (12.0)	107 (66.1)	0 (0)
Having both CWP and CMDs	116 (1.5)	15 (9.3)	0 (0)
**Outcomes**			
Disability pension	1242 (15.8)	35 (21.6)	25 (15.4)
Unemployment (> 90 days)	1447 (18.4)	34 (21.0)	33 (20.4)
All-cause mortality	417 (5.3)	11 (6.8)	2 (1.2)

* *CWP* Chronic Widespread pain, *CMDs* Common mental disorders

### Disability pension

In the whole sample with a history of SA, having CWP and/or CMDs were associated with a higher risk for DP compared to those without CWP and CMDs in the crude model. In Model 1, we adjusted for socio-demographic factors including sex, age, education, children living at home, type of living area and cohort effect and the HRs for DP showed somewhat of changes (range of HRs: 1.54–2.84), indicating some influence from socio-demographic factors on the associations. The increased risk of DP remained even after additional control for health-related factors (i.e. self-rated health, pain, headache, migraine, and cancer), particularly for those having only CWP (HR: 2.43, 95% CI: 2.03–2.93) and both CWP and CMDs (HR: 2.65, 95% CI: 1.89–3.71). In the analyses of discordant twin pairs, the HR was lower and became non-significant for those who only had CMDs (HR: 0.96, 95% CI: 0.52–1.81) compared to the estimate of the whole sample. This result suggests that familial factors influence the association between CMDs and all-cause DP (Table 2).

**Table 2:** Hazard ratios with 95% confidence intervals for the associations between chronic widespread pain, common mental disorders and disability pension in the whole sample (*n* = 7884) and of the co-twins discordant for exposure (*n* = 324)

	Disability pension			
	Crude	Model 1^**a**^	Model 2^**b**^	Co-twin model^**c**^
**CWP and/or CMDs**				
No CWP and CMDs	1	1	1	1
Having CWP but no CMDs	2.93 (2.44–3.52)	2.49 (2.07–2.99)	2.43 (2.02–2.93)	2.98 (1.59–5.58)
No CWP but having CMDs	1.22 (1.03–1.44)	1.54 (1.30–1.82)	1.50 (1.27–1.78)	0.96 (0.52–1.81)
Having both CWP and CMDs	2.49 (1.78–3.47)	2.84 (2.03–3.97)	2.65 (1.89–3.71)	1.39 (0.42–4.59)

* *CWP* Chronic Widespread pain, *CMDs* Common mental disorders

^a^ Model 1: adjusted for sex, age, education, children living at home, type of living area, and cohort

^b^ Model 2: adjusted for sex, age, education, children living at home, type of living area, cohort, self-rated health, pain, headache, migraine, and cancer

^c^ Co-twin model: adjusted for age and familial (genetic and shared environmental) factors by matching

### Unemployment

An association between CMDs and subsequent unemployment > 90 days was found in the crude model as well as (HR: 1.38, 95% CI: 1.19–1.60) after adjustment for all the covariates (HR: 1.36, 95% CI: 1.17–1.58). The HRs changed slightly after controlling for the covariates, meaning a minor influence from those factors on the associations between CMDs and unemployment > 90 days. The HR was attenuated in the co-twin model (HR: 1.14, 95% CI: 0.67–1.92), which suggest that the association between CMDs and unemployment was influenced by familial factors (Table 3).

**Table 3:** Hazard ratios with 95% confidence intervals for the associations between chronic widespread pain, common mental disorders and unemployment (> 90 days) in the whole sample (*n* = 7884) and of the co-twins discordant for exposure (*n* = 324)

	Unemployment (> 90 days)			
	Crude	Model 1^**a**^	Model 2^**b**^	Co-twin model^**c**^
**CWP and/or CMDs**				
No CWP and CMDs	1	1	1	1
Having CWP but no CMDs	1.10 (0.85–1.42)	1.15 (0.89–1.48)	1.09 (0.84–1.41)	0.77 (0.32–1.85)
No CWP but having CMDs	1.38 (1.19–1.60)	1.40 (1.21–1.62)	1.36 (1.17–1.58)	1.14 (0.67–1.92)
Having both CWP and CMDs	1.38 (0.92–2.07)	1.32 (0.88–1.97)	1.18 (0.79–1.78)	1.53 (0.54–4.33)

* *CWP* Chronic Widespread pain, *CMDs* Common mental disorders

^a^ Model 1: adjusted for sex, age, education, children living at home, type of living area, and cohort

^b^ Model 2: adjusted for sex, age, education, children living at home, type of living area, cohort, self-rated health, pain, headache, migraine, and cancer

^c^ Co-twin model: adjusted for age and familial (genetic and shared environmental) factors by matching

### Mortality

Table 4 shows HRs for all-cause mortality in the whole sample with a history of SA and among twins discordant for the exposures. Having either CWP or CMDs was associated with a higher risk for all-cause mortality after controlling for all the covariates (Having only CWP: HR: 1.95, 95% CI: 1.39–2.74; Having only CMDs: HR: 1.60, 95% CI: 1.19–2.16). The HRs increased more after controlling for socio-demographic factors and cohort effects compared to the model controlled with additional health-related factors. This indicates that socio-demographic factors and cohort effects had more impact on the associations between CWP and/or CMDs and mortality than health-related factors. In the analyses of discordant twins, the HR increased, especially for those only with CMDs. This may indicate a direct (i.e. not affected by familial confounding) link between CMDs and all-cause mortality.

**Table 4:** Hazard ratios with 95% confidence intervals for the associations between chronic widespread pain, common mental disorders and all-cause mortality in the whole sample (*n* = 7884) and of the co-twins discordant for exposure (*n* = 324)

	Mortality			
	Crude	Model 1^**a**^	Model 2^**b**^	Co-twin model^**c**^
**CWP and/or CMDs**				
No CWP and CMDs	1	1	1	1
Having CWP but no CMDs	1.89 (1.35–2.65)	1.93 (1.38–2.72)	1.95 (1.39–2.74)	3.15 (0.44–22.47)
No CWP but having CMDs	1.27 (0.94–1.70)	1.58 (1.17–2.13)	1.60 (1.19–2.16)	8.09 (1.74–37.53)
Having both CWP and CMDs	1.43 (0.68–3.02)	1.83 (0.86–3.88)	1.83 (0.86–3.88)	Too few

* *CWP* Chronic Widespread pain, *CMDs* Common mental disorders

^a^ Model 1: adjusted for sex, age, education, children living at home, type of living area, and cohort

^b^ Model 2: adjusted for sex, age, education, children living at home, type of living area, cohort, self-rated health, pain, headache, migraine, and cancer

^c^ Co-twin model: adjusted for age and familial (genetic and shared environmental) factors by matching

### Sensitivity analyses

Sensitivity analyses were conducted for DP, long-term unemployment and mortality among all twins and twins discordant for the exposure after excluding neck-, back-, and shoulder pain, migraine, and cancer at baseline (data not shown). The results of the sensitivity analyses were of the same magnitude and direction as the main analyses which included pain sites, migraine, and cancer.

## Discussion

In this population-based prospective study of 7884 twin individuals with a history of SA we found that individuals with either CWP or CMDs had an elevated risk of subsequent DP and all-cause mortality after controlling for socio-demographic and health factors. Moreover, having both CWP and CMDs increased the risk of DP while individuals who only had CMDs had a higher risk of long-term unemployment of more than 90 days compared to individuals without CWP and CMDs. Familial factors influenced the associations between CMDs and future DP and long-term unemployment.

Epidemiological studies suggest that a bidirectional relationship exists between CWP and CMDs [27] and the negative health consequences of CWP and CMDs are numerous [4, 28]. For example, individuals experiencing CWP or CMDs often report poor health-related quality of life, psychosomatic symptoms and work disability [29, 30]. The association between CWP and CMDs with SA and DP has seldom been investigated but some studies have reported negative consequences with respect to work status [7, 31]. A Swedish population-based study has shown that chronic pain in age groups below the age of 65 was strongly associated with a lower prevalence of working [31]. Another previous study also reported that individuals having either or both of CWP and CMDs are less likely to be participating in the labour market [7]. The findings in the current study agree with the existing research. We also found that having CWP but not CMDs was associated with a comparable risk of future DP compared to individuals having both CWP and CMDs. This result might indicate that among individuals with a history of SA, CWP seems to play a more important role than CMDs in transition from temporary work disability to permanent work disability. However, undetected mental disorders are possible among individuals with CWP. In addition, besides impaired physical and mental health, transition to DP involves a number of other factors, such as discrimination against workers with health problems on the labour market and social marginalisation [32]. Hence, future studies should include such factors.

On the other hand, we found that CMDs was a risk factor for unemployment for those with SA, which is in line with previous studies, showing that individuals with CMDs had higher levels of unemployment [30, 33]. This may reflect the deteriorating levels of functioning of individuals with anxiety and depression together with few possibilities to adjust the work situation, contributing to work disability and exit from the labour market [34, 35]. Additionally, familial factors seem to influence the associations between CMDs and subsequent DP and unemployment. Previous twin studies have recognised the influence of genetic factors on CMDs [13, 14] as well as on DP [16, 17] and our findings suggest that the associations between CMDs and work disability and labour market participation, is also partially explained by genetics. Still, a large number of studies have reported the importance of environmental factors such as work environment in the association between CMDs and work disability [36, 37]. For CWP, environmental factors such as occupational factors also seem important for the risk of DP and unemployment.

Moreover, CWP and CMDs were independently associated with increased mortality risk, which is consistent with previously conducted studies [4, 38]. Increased risk of premature death, both from natural and unnatural causes, has been reported for CWP and CMDs and explanations for this include poorer quality of medical care, smoking, unhealthy diet, and a generally unhealthy lifestyle [39]. Also, excess mortality may reflect availability and quality of health care. Our results do not only require attention for the vulnerable individuals, but also underline the increased societal costs for health care, welfare benefits and productivity loss. However, we did not observe a higher risk of mortality among individuals experiencing both CWP and CMDs. Due to the low prevalence of people with both CWP and CMDs in the study, this may be why we were unable to find statistically significant results with mortality. It may also be explained by the fact that comorbid conditions of CWP and CMDs often require more medical treatment than presenting a single symptom, which may contribute to lowering the likelihood of early mortality [40].

### Strengths and limitations

Strengths of this study include the large population-based sample of twins and the use of Swedish nationwide register data of high quality [41], which reduced the risk of loss to follow-up and recall bias. Moreover, we could include discordant twin pairs in the analyses, which enable to control for the influence from familial factors. However, some analyses were still underpowered with broad CIs. In this study, we were also able to include several relevant covariates in relation to the exposure and outcomes. Still, there might be other factors than those included here that are associated with the outcomes, such as unhealthy lifestyle (i.e. smoking behaviour, alcohol consumption, lack of physical activity) etc.

The limitations of the study include that data on sick-leave spells < 14 days were not available for employed individuals. Thus, the findings from the study may not be generalizable to individuals with short-term SA spells (< 14 days). In addition, since the survey was extensive and included many questions, there are some internal missing data. This might lead to underestimation of the results. Also, it was not possible to perform analyses with only same-sex discordant twins due to low numbers, or analyses stratified on zygosity, making further investigation of familial factors impossible. Moreover, the co-twin models lacked power and findings from these analyses needs to be interpreted with caution. Moreover, because our measures of CWP and CMDs relied on self-reports and the diagnostic criteria for CWP has changed over time [42], it is likely to have some misclassification. However, misclassification is possible to be non-differential in MZ and DZ twins and it is therefore not likely that our estimation of the influence of familial factors is biased. Our measure of CWP was not based on clinical examination and thus inferences should be with caution when our findings are compared with studies based on clinical examination. On the other hand, questionnaire- and interview-based screening have shown relatively high positive predictive value and test-retest reliability [43] and these are the only realistic means to gather data over ten of thousands individuals in population-based samples. The questionnaire and interview data for measuring CWP and CMDs were collected during 1998–2006. Hence, a new collection of data in this field is recommended for facilitating future studies with updated findings. In addition, the Swedish government launched stricter rules for granting DP since 2008, which might result in that individuals with DP had a higher medical severity than before these regulations were implemented [11]. Our measures of CWP and CMDs were self-reported, hence they may or may not require any medical attention which could lead to underestimated risk of DP as the severity and chronicity of CWP and CMDs is unknown. Still, the findings in this study might be generalised to working-aged individuals living in countries with comparable social insurance systems.

## Conclusions

To conclude, this population-based prospective twin study showed that CWP was associated with an elevated risk of DP and mortality among individuals with a history of SA. Having both CWP and CMDs was also associated with a higher risk of DP. Moreover, CMDs was an independent risk factor for mortality while familial factors influence the associations between CMDs and future DP and unemployment.

## Supplementary information

**Additional file 1:** **Figure S1.** Proportional hazards assumption test with log minus log plot for the association between CWP and/or CMDs and disability pension. **Figure S2.** Proportional hazards assumption test with log minus log plot for the association between CWP and/or CMDs and unemployment. **Figure S3.** Proportional hazards assumption test with log minus log plot for the association between CWP and/or CMDs and mortality.

## Data Availability

The authors are not allowed to make the micro-level data used in this study publicly available, due to their sensitive nature. According to the General Data Protection Regulation, the Swedish law SFS 2018:218, the Swedish Data Protection Act, the Swedish Ethical Review Act, and the Public Access to Information and Secrecy Act, these type of sensitive data can only be made available after legal review, for researchers who meet the criteria for access to this type of sensitive and confidential data. Readers may contact Associate Professor Pia Svedberg (pia.svedberg@ ki.se) regarding the data.

## Abbreviations

CI: Confidence Interval
CMDs: Common mental disorders
CWP: Chronic widespread pain
DP: Disability pension
DSM-IV: Diagnostic and Statistical Manual of Mental Disorders, Fourth Edition
DZ: Dizygotic
HR: Hazard Ratios
LISA: Longitudinal Integration Database for Health Insurance and Labour Market Studies
MiDAS: MicroData for Analysis of the Social Insurance database
MZ: Monozygotic
SA: Sickness absence
SALT: Screening Across the Lifespan Twin Study
STAGE: Study of Twin Adults - Genes and Environment
STODS: Swedish Twin project of Disability pension and Sickness absence
STR: Swedish Twin Registry
