# Supplementary material for: Adverse outcomes of chronic widespread pain and common mental disorders in individuals with sickness absence – a prospective study of Swedish twins
Source: BMC Public Health. 2020 Aug 27;20:1301. doi: 10.1186/s12889-020-09407-9 (PMC7457303; doi:10.1186/s12889-020-09407-9)
Supplement: Supplementary file 1 — Additional file 1: Figure S1. Proportional hazards assumption test with log minus log plot for the association between CWP and/or CMDs and disability pension. Figure S2. Proportional hazards assumption test with log minus log plot for the association between CWP and/or CMDs and unemployment. Figure S3. Proportional hazards assumption test with log minus log plot for the association between CWP and/or CMDs and mortality. [file 12889_2020_9407_MOESM1_ESM.docx]

**Supplementary materials**


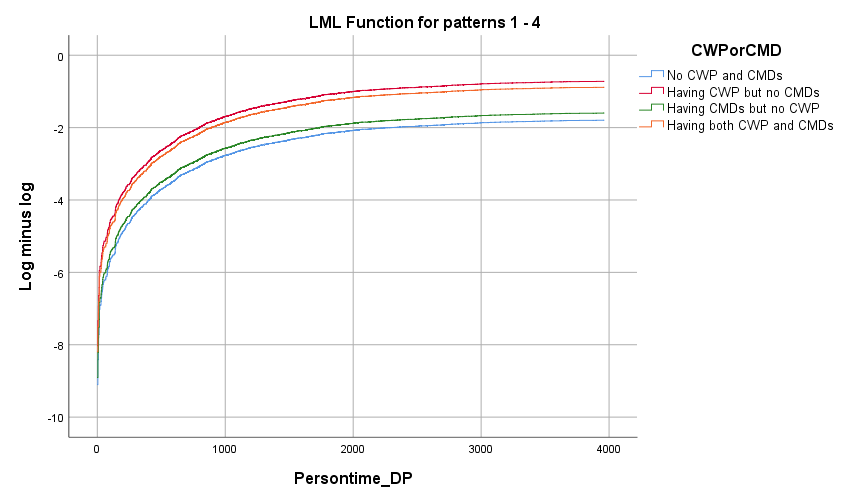


**Figure 1. Proportional hazards assumption test with log minus log plot for the association between CWP and/or CMDs and disability pension.**


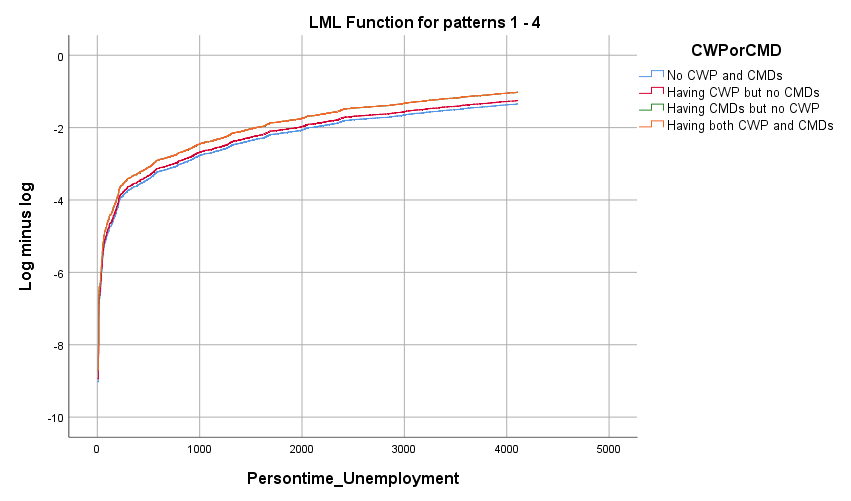


**Figure 2. Proportional hazards assumption test with log minus log plot for the association between CWP and/or CMDs and unemployment.**


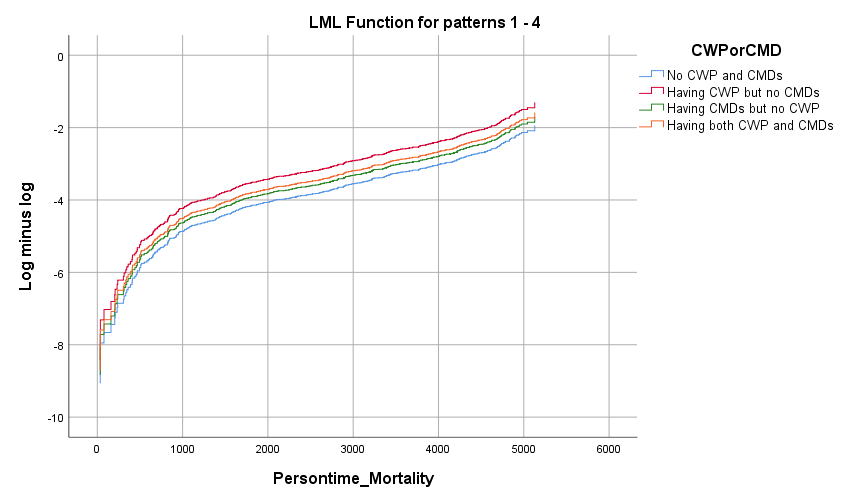


**Figure 3. Proportional hazards assumption test with log minus log plot for the association between CWP and/or CMDs and mortality.**
